# Clinical Relevance and Characteristics of *Aspergillus calidoustus* and Other *Aspergillus* Species of Section *Usti*

**DOI:** 10.3390/jof6020084

**Published:** 2020-06-12

**Authors:** Emmanouil Glampedakis, Véronique Erard, Frederic Lamoth

**Affiliations:** 1 Infectious Diseases Service, Lausanne University Hospital and University of Lausanne, 1011 Lausanne, Switzerland; emmanouil.glampedakis@chuv.ch; 2 Clinique de Médecine et spécialités, infectiologie, HFR-Fribourg, 1708 Fribourg, Switzerland; Veronique.Erard@h-fr.ch; 3 Institute of Microbiology, Lausanne University Hospital and University of Lausanne, 1011 Lausanne, Switzerland

**Keywords:** *Aspergillus ustus*, *Aspergillus pseudodeflectus*, *Aspergillus granulosus*, *Aspergillus insuetus*, *Aspergillus puniceus*, *Aspergillus keveii*, invasive aspergillosis

## Abstract

The *Aspergilli* of section *Usti* (group *ustus*) are represented by over 20 species, of which *Aspergillus calidoustus* is the most relevant human pathogen. Invasive aspergillosis (IA) caused by these fungi is rare but could represent an emerging issue among the expanding population of patients with long-term immunosuppression receiving antifungal prophylaxis. Clinicians should be aware of this unusual type of IA, which often exhibits distinct clinical features, such as an insidious and prolonged course and a high occurrence of extra-pulmonary manifestations, such as skin/soft tissue or brain lesions. Moreover, these *Aspergillus* spp. pose a therapeutic challenge because of their decreased susceptibility to azole drugs. In this review, we outline the microbiological and clinical characteristics of IA due to *Aspergillus* spp. of section *Usti* and discuss the therapeutic options.

## 1. Introduction

Fungi of the genus *Aspergillus* represent the most important pathogenic molds for humans, causing invasive aspergillosis (IA) in patients with impaired immune defenses. While over 300 *Aspergillus* spp. have been described, the vast majority of IA cases are attributed to less than five species, consisting mainly of *A. fumigatus* (60–80% cases) and *A. flavus*, *A. niger* (or related cryptic species) and *A. terreus* for most of the remaining cases [1,2,3,4]. A recent study however suggested that epidemiology of IA may evolve as a consequence of the widespread use of anti-mold azole prophylaxis (i.e., posaconazole or voriconazole) with emergence of *Aspergillus* of section *Usti* (group *ustus*) exhibiting natural resistance to these antifungals [5]. This section includes over 20 species that are ubiquitous molds found in the indoor and outdoor environment [6]. Notably, they were the most frequent *Aspergillus* spp. found in drinking water distribution systems in Norway, including from hospital tap water [7]. *A. ustus* and related species were also frequently recovered from water-damaged buildings and from caves affected by human activities [8,9]. While the first case of IA due to *Aspergillus* of section *Usti* was described in 1974 [10], these infections have been increasingly reported in the literature since 2000 [11,12].

The aim of this review is to provide a practical summary of what infectious diseases specialists and microbiologists should know about *Aspergillus* spp. of section *Usti* for their daily practice.

## 2. Taxonomy and Microbiology

Based on phylogenetic analyses, there are currently 26 recognized *Aspergillus* species belonging to section *Usti* (Table 1) [6,8,13]. Most of them, including *A. ustus* sensu stricto, are unable to grow at 37 °C and therefore are not considered as human pathogens. Actually, most cases of human infections that were attributed to *A. ustus* in the literature, were secondarily reassigned to a novel distinct species, *A. calidoustus*, which is able to grow at 37 °C [14]. Two other closely related species, *A. pseudodeflectus* and *A. granulosus*, are also thermotolerant at human body temperature and were also found to be able to cause invasive infections in humans [11,15,16,17]. *A. ustus* sensu stricto was also isolated from a patient with aspergillosis localized to the skin and soft tissue [11]. Other non-thermotolerant species of section *Usti* that were isolated as colonizers or contaminants from clinical specimens include *A. insuetus*, *A. puniceus* and *A. keveii* [11].

Morphological characteristics of *Aspergillus* spp. of section *Usti* are usually reliable for identification at the section level. However, species identification would require partial sequencing of the beta-tubulin (*BenA*) or calmodulin (*CaM*) genes, which is not routinely available in most diagnostic microbiology laboratories [11]. Standard sequencing methods targeting the internal transcribed spacer (ITS) or 26-28S rDNA are not reliable enough for identification beyond the section level. Experience with matrix-assisted laser desorption ionization–time of flight mass spectrometry (MALDI-TOF MS) is limited for these rare species and misidentification has been reported [19].

**Figure 1:**
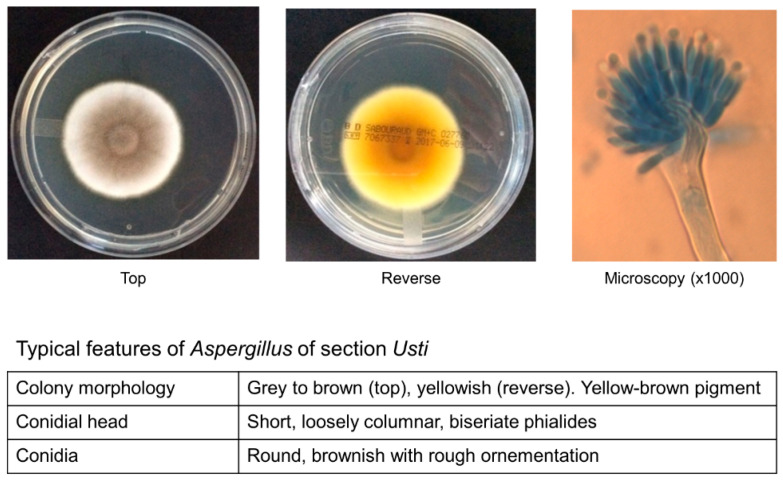
Morphological aspects of *Aspergillus calidoustus.* Macroscopic aspect of the colony on Sabouraud dextrose agar medium, top (**left**) and reverse (**middle**). Microscopic aspect (1000 ×) of a conidial head (staining: lactophenol blue) (**right**). Note: rough ornamentation of conidia is not visible here and could better visualized by scanning electron microscopy.

Most species of section *Usti* will grow at 25–30 °C. Higher temperature (37 °C) allows distinguishing *A. calidoustus* or other thermotolerant species (e.g., *A. pseudodeflectus*, *A. granulosus*) from nonpathogenic species. Colonies are usually apparent between 2 and 5 days of growth. Macroscopic aspects on standard fungal culture media (e.g., Czapek yeast extract agar, Sabouraud or potato dextrose agar) show velvety greyish to brown cinnamon colonies (Figure 1, left) [6,20]. The yellowish reverse color with presence of yellow-brown soluble pigment is typical but can be absent for some species and/or according to the culture medium (Figure 1, middle). Under the microscope, conidial heads are usually short and loosely columnar with biseriate phialides (Figure 1, right) [6,20]. Conidia typically harbor rough ornamentation. Some specific characteristics (Hülle cells, Ehrlich reaction, growth on creatine, production of extrolites) may help distinguishing the different species, but these methods require the expertise of reference laboratories [6,20].

Antifungal susceptibility testing shows very similar profiles across species [11,21]. Amphotericin B is the most active drug in vitro with minimal inhibitory concentration encompassing 50% (MIC_50_) and 90% (MIC_90_) isolates of 0.5 and 1 µg/mL, respectively [11]. Azoles exhibit in vitro activity at concentrations that are usually at the upper limit or beyond the therapeutic range of concentration. Notably, isavuconazole displays somewhat higher activity compared to voriconazole and posaconazole (this latter one being the less active): MIC_50_/MIC_90_ of 2/4 µg/mL, 8/8 µg/mL and 16/>16 µg/mL, respectively [11]. The fungistatic activity of echinocandins is comparable to that against other *Aspergillus* spp. with micafungin and anidulafungin exhibiting lower MIC compared to caspofungin [21]. The novel long-lasting echinocandin rezafungin (CD101) and the glucan synthase inhibitor ibrexafungerp (SCY-078) are also active against *Aspergillus* of section *Usti* [22,23]. Terbinafine has good in vitro activity (MIC 0.25 to 1 µg/mL), and its combination with voriconazole was synergistic in vitro and in a *Galleria mellonella* model of infection [24].

## 3. Epidemiology and Clinical Characteristics

IA caused *by Aspergillus* section *Usti* (further referred as *A. ustus* IA) remains a rare disease. In a cohort of 218 culture positive IA from the Transplant-Associated Infection Surveillance Network (TRANSNET), *A. ustus* complex species were the fifth cause of IA being responsible for 2.7% of all cases [25]. In the Prospective Alliance Therapy (PATH) registry, these species accounted for 0.8% of cases (rank 6th) [4]. In a single center study of 24 microbiologically documented breakthrough invasive mold infections, *A. ustus* accounted for 12.5% of all episodes and 43% of IA [5]. Outbreaks of *A. ustus* IA have been reported among hematopoietic stem cell transplant (HSCT) or solid-organ transplant (SOT) recipients [26,27]. While these data are mainly derived from North American cohorts, the incidence of *A. ustus* IA in other regions of the world is not well described.

The largest epidemiological description of proven/probable *A. ustus* IA included 72 cases, of which 45 were obtained from previous published case reports or small case-series (1974–2018) and 27 were collected via a screening of microbiological databases of 22 European hospital centers (2007–2018) [11]. Most patients were non-neutropenic transplant recipients (47% HSCT and 33% SOT recipients) receiving long-term immunosuppressive therapy (anti-calcineurin drugs and/or corticosteroids). About half of them (47%) had ongoing anti-mold azole prophylaxis (mainly posaconazole) at time of diagnosis. This observation is consistent with the above mentioned epidemiological studies suggesting a higher prevalence of *A. ustus* IA among transplant patients and those receiving anti-mold azole prophylaxis [5,26,27].

*A. ustus* IA were disseminated (i.e., more than one organ affected) in 33% cases. While the lung was affected in 76% cases, primary or secondary extra-pulmonary sites of infections were frequently observed. Skin and/or soft-tissue lesions were present in 28% cases and cerebral aspergillosis in 14% cases [11]. Serum galactomannan was positive in 85% patients. Overall mortality was high (58% at 6 months, with IA being considered as a major or partial cause of death in 81% of cases) [11].

In summary, *A. ustus* IA exhibit some distinct clinical features compared to other IA, as they seem to affect mainly non-neutropenic transplant patients receiving anti-mold active prophylaxis and have a propensity to cause primary or secondary skin lesions or other extra-pulmonary foci of infection. The clinical case presented in Box 1 is illustrative of these characteristics and shows the insidious course of this fungal disease with notably the positive galactomannan in serum preceding the clinical signs of infection by several weeks or months.

Box 1 **Illustrative case of *Aspergillus calidoustus* invasive aspergillosis.**

A 64-year old woman underwent allogeneic hematopoietic stem cell transplantation for acute myeloid leukemia. Three years later, she was treated by two chemotherapy cycles (FLAG and FLAG-IDA) for two consecutive relapses of the hematologic cancer, followed by maintenance therapy with azacitidine and sorafenib. She was receiving tacrolimus and corticosteroids for cutaneous and digestive graft versus host disease (GVHD). Antifungal prophylaxis with posaconazole was administered with appropriate trough concentrations (>0.5 mg/L). During follow-up, an increase in serum galactomannan was observed with a first positive value at 1.8 (optical density index), while she was asymptomatic. Three months later, she noticed painless skin nodules on her right leg, upper back and axillary hollow. Serum galactomannan at this time was persistently positive (6.43). Histopathological examination of the nodules revealed subcutaneous granulomas with mycelial elements. Cultures of skin biopsy grew a mold identified as an *Aspergillus* group *ustus* by sequencing of the 26-28S rDNA and identified at species level as *Aspergillus calidoustus* by partial sequencing of the beta-tubulin (*BenA*) and calmodulin (*CaM*) genes. Total body CT and ^18^F-FDG PET/CT did not reveal any other lesion.

The patient received multiple antifungal treatment lines (liposomal amphotericin B with caspofungin, voriconazole with terbinafine, liposomal amphotericin B with caspofungin and terbinafine). Following surgical excision of all skin nodules, she experienced a recurrence of infection with suspected fungal arthritis of the right shoulder, which was treated by intra-articular injections of amphotericin B. A reduction of the immunosuppressive regimen was attempted, but the patient experienced a flare of GVHD and ultimately died. While all clinical foci of infection had resolved, serum galactomannan was persistently positive at time of death. Autopsy however did not reveal evidences of remaining invasive mold infection.

## 4. Treatment

As previously mentioned, the species of *Aspergillus* section *Usti* exhibit high MICs to the azole drugs, which represent the first-line antifungal therapy of IA [28]. As a result, current guidelines recommend the use of amphotericin B lipid formulations, which are the most active drug in vitro [28]. In practice, antifungal management is difficult with frequent use of multiple antifungal agents, either consecutively or in combination (Box 1) [11]. Interestingly, our analysis of the 72 *A. ustus* IA cases show that voriconazole was used as first-line therapy (i.e., first antifungal drug administered for at least 10 consecutive days) in a substantial proportion of cases [11]. These patients actually seemed to be less immunocompromised (non-HSCT recipients) and less severely ill with IA that were non-disseminated and classified as probable only, in comparison to those who were treated by amphotericin B. Not surprisingly, the mortality rate was significantly lower in this subgroup compared to amphotericin B-treated patients. Because of these evident biases in retrospective non-matched cohorts, it is not possible to draw conclusions about comparative drug efficacy. Nonetheless, it is noteworthy that voriconazole and amphotericin B were equally effective in a *Galleria mellonella* model of *A. calidoustus* infection [24]. The novel triazole isavuconazole seems to be somewhat more active than voriconazole in vitro, but clinical experience with this drug for the treatment of *A. ustus* IA is still very limited [29].

The potential benefit of drug combination is also debated. Indeed, an echinocandin, in combination with amphotericin B or voriconazole, was part of the first-line antifungal regimen in about one third of cases [11]. Some patients also received a combination of amphotericin B and voriconazole. Overall, mortality was high among patients receiving combination therapies, which may actually reflect the severity of the initial presentation of the disease in these cases. In vitro, these drug combinations were classified as indifferent [24]. Only the combination of voriconazole and terbinafine demonstrated a synergistic interaction in vitro and in the *Galleria* model [24]. While clinical experience with terbinafine for invasive mold infections is very limited, this drug may have an interest as adjunctive treatment for *A. ustus* IA because of its high penetration in skin and soft tissue and possibly in the brain [30]. Similarly, the combination of voriconazole and terbinafine has been used for other refractory mold diseases, such as scedosporiosis, although its benefit was not demonstrated [31].

From these observations, we can conclude that the optimal therapeutic approach of *A. ustus* IA would still deserve further investigations. Notably, this is another example that in vitro data do not necessarily correlate with clinical efficacy, as it has been previously shown for other difficult-to-treat mold infections [32]. Non-pharmacological parameters, such as recovery of the immune system or the initial severity of the disease with delay in diagnosis may represent the predominant predictors for outcome.

Our personal approach of *A.ustus* IA, as described in Table 2, is to consider liposomal amphotericin B as the first-line treatment, especially for severe cases. However, we consider that voriconazole or isavuconazole (this latter drug being even more active in vitro) alone or combined with terbinafine remain possible therapeutic options, in particular for less severe cases (localized and/or probable IA in patients with mild/moderate immunosuppression and in the absence of previous mold-active azole prophylaxis) or as second-line therapy in case of nephrotoxicity of amphotericin B or for maintenance therapy. Posaconazole should be avoided because of its quasi-lack of in vitro activity and the occurrence of breakthrough *A. ustus* IA with this drug. The role of echinocandins remains unclear, but this drug class could be used as adjunctive therapy in severe cases.

Novel broad-spectrum antifungal agents are needed to treat *A. ustus* and other refractory mold infections. Some of them provided promising in vitro results. The Gwt1p inhibitor APX001A (E1210) and olorofim (F901318), an inhibitor of pyrimidine biosynthesis, show good activity against *Aspergillus* species of section *Usti* [33,34].

## 5. Conclusions

*Aspergillus* of section *Usti* (group *ustus*), in particular *A. calidoustus*, are increasingly recognized as causal agents of IA, as a possible consequence of the extent of the population of transplant patients with long-term immunosuppression and the widespread use of antifungal prophylaxis. This mold infection is challenging because of its insidious course, atypical presentation and multidrug resistance. Clinicians should suspect *A. ustus* IA in front of a transplant patient with persistently positive galactomannan despite no clinical or radiological evidence of IA or in the presence of skin lesions or soft tissue nodules. Optimal antifungal therapy still needs to be better defined. While amphotericin B is the most active drug in vitro, other antifungals or drug combinations (e.g., voriconazole or isavuconazole +/− terbinafine, adjuvant echinocandin) could be considered in selected situations.

## Figures and Tables

**Table 1:** The 26 *Aspergillus* species of section *Usti* and their pathogenic role in humans.

Isolated in Clinical Specimens	Environmental Samples Only	
**Proven/probable IA ^1^ cases** *A. calidoustus* ^2^ *A. pseudodeflectus* ^3^ *A. granulosus* ^4^ *A. ustus* ^5^	*A. amylovorus* *A. asper* *A. baeticus* *A. californicus* *A. carlsbadensis* *A. cavernicola* *A. collinsii* *A. deflectus* *A. egyptiacus* *A. elongatus*	*A. turkensis* *A. germanicus* *A. heterothallicus* *A. kassunensis* *A. lucknowensis* *A. monodii* *A. pseudoustus* *A. subsessilis* *A. thessauricus*
**Colonization only** *A. insuetus* *A. keveii* *A. puniceus*		

IA: invasive aspergillosis. ^1^ Proven probable invasive aspergillosis according to the criteria of the European Organization for Research and Treatment of Cancer (EORTC) and Mycoses Study Group (MSG) [18]. ^2^ Major cause of IA in humans [11]. ^3^ Three reported cases of probable IA [11,15]. ^4^ Two reported cases of proven IA [16,17]. ^5^ Single reported case of proven soft tissue IA [11].

**Table 2:** Current antifungal therapeutic options against *Aspergillus calidoustus* and other *Aspergillus* spp. of section *Usti.*

Antifungal Drug Classes	Evidences	Comments
Amphotericin B	Relatively good in vitro activity (MIC 0.25–2 µg/mL) [11,21] Effective in a *Galleria* model [24]	Recommended as first-line on the basis of optimal in vitro activity (use lipid-based formulation)
Mold-active azoles	Relatively low in vitro activity (MIC 2 – 16 µg/mL): isavuconazole > voriconazole > posaconazole [11,21] Voriconazole effective in a *Galleria* model of infection [24] Caveat: breakthrough infections frequently reported (mainly under posaconazole, but also voriconazole)	Pre-clinical and clinical data suggest possible use in selected situations (e.g., less severe cases or second-line/maintenance treatment, absence of previous mold-active azole prophylaxis) Avoid posaconazole
Echinocandins	Fungistatic effect: micafungin/anidulafungin > caspofungin [21]	May be used in combination with either amphotericin B or triazoles despite no evidence of synergism Few experience as monotherapy, use only if no other alternatives (preferably micafungin or anidulafungin)
Terbinafine	Relatively good in vitro activity (MIC 0.25–1 µg/mL) [21,24] Effective in a *Galleria* model of infection [24] In vitro and in vivo (*Galleria*) synergism with voriconazole, posaconazole and isavuconazole [24] In vitro antagonism with amphotericin B [24] Accumulation in skin (no sustained levels in blood) [30]	May be combined with voriconazole (or isavuconazole) in selected situations (see above, possible interest in patients with skin lesions or alternative to amphotericin B in case of intolerance) Use as monotherapy not recommended

MIC: minimal inhibitory concentration, >: activity superior to.
